# Pan-biological characteristics of ataxin-2 protein

**DOI:** 10.4103/NRR.NRR-D-25-00751

**Published:** 2025-11-25

**Authors:** André Conceição, Clévio Nóbrega

**Affiliations:** Algarve Biomedical Center Research Institute (ABC-RI), Faro, Portugal; PhD Program in Biomedical Sciences, Faculdade de Medicina e Ciências Biomédicas, Universidade do Algarve (UAlg), Faro, Portugal; Faculdade de Medicina e Ciências Biomédicas, Universidade do Algarve (UAlg), Faro, Portugal

**Figure 1:**
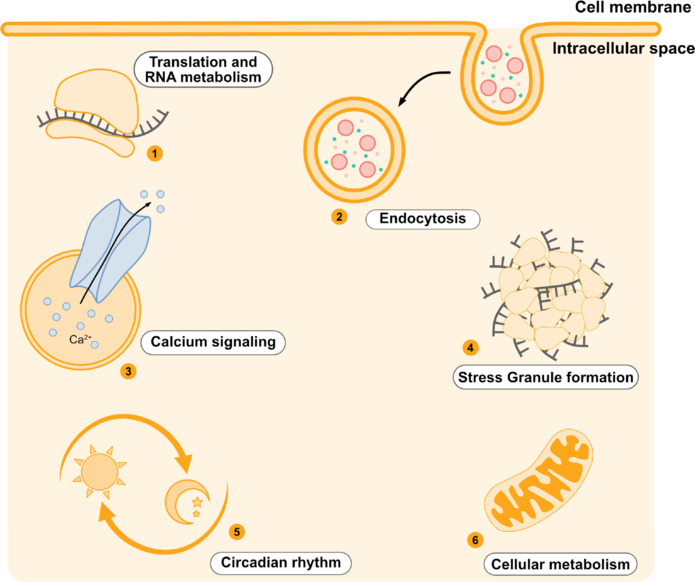
Pan-biological characteristics of ataxin-2 protein. Ataxin-2 is a ubiquitously expressed protein with multiple functions. Ataxin-2 has been described to play roles in diverse biological processes crucial for normal cellular function, including (1) translation and RNA metabolism, (2) endocytosis, (3) calcium signaling, (4) stress granule formation, (5) circadian rhythm, and (6) cellular metabolism.

Ataxin-2 is a 140 kDa cytoplasmic multifunctional protein that plays fundamental roles in diverse cellular mechanisms (Costa et al., 2024). Although widely studied in the context of the neurodegenerative diseases spinocerebellar ataxia type 2 (SCA2) and amyotrophic lateral sclerosis (ALS), ataxin-2 functions span far beyond its pathogenic properties in the disease context (**Figure 1**). In fact, it has a wide range of biological functions that include RNA metabolism, translation regulation, stress response, endocytosis, calcium signaling, and the control of circadian rhythm. In this perspective, we highlight the main roles of ataxin-2 protein, which are described in detail in Costa et al. (2024).

Ataxin-2 is encoded by the *ATXN2* gene and is expressed ubiquitously across tissues, with particular abundance in the central nervous system, especially in the cerebellar Purkinje cells (Pulst et al., 1996; Costa et al., 2024). At a structural level, ataxin-2 is composed by 1312 amino acids and 6 protein domains that include: two proline-rich Src homology 3 (SH3) domain binding motifs — i) SBM1 and ii) SBM2; (iii) a polyglutamine (polyQ) tract (22 glutamines most common); (iv) a Like Sm domain (Lsm); (v) a Lsm-associated domain; (vi) poly(A)-binding protein 1 (PABP-1) interacting motif 2 (PAM2) The SBM domains are predicted to interact with endophilins, suggesting a role in mechanisms of endocytosis (Costa et al., 2024). The Lsm region is involved in the metabolism of mRNAs (Costa et al., 2024). The Lsm-associated domain is responsible for ataxin-2 exportation from the endoplasmic reticulum (Huynh et al., 2003). Finally, the PAM2 motif modulates protein translation (Nóbrega et al., 2015; Costa et al., 2024).

Ataxin-2 is an RNA-binding protein that directly binds RNA. Ataxin-2 is thought to be involved in multiple steps of RNA metabolism, including RNA splicing, stabilization, and translation (Costa et al., 2024). Ataxin-2 splicing activity is suggested to occur through the interaction with A2BP1, RNA-binding motif protein 9, and the RNA-binding protein with multiple splicing (Lim et al., 2006). Moreover, ataxin-2 can increase the stability of mRNAs by interacting with their 3’UTR region. Additionally, ataxin-2 is thought to inhibit the poly(A) nuclease complex, thereby preserving mRNA polyadenylation and promoting transcript availability (Costa et al., 2024).

Ataxin-2 is also suggested to play a role in the formation of stress granules (SGs), a structure that is formed in response to cellular stress, crucial for restoring cell homeostasis in deleterious situations (Marcelo et al., 2021a). Ataxin-2 is a component of SGs, and recent studies position ataxin-2 as a central player in the nucleation of SGs (Nunes et al., 2019). During a cellular stress condition, PABPC1-bound to untranslated poly(A)-mRNA recruits ataxin-2, forming the PABPC1:mRNA:ataxin-2 complex. The increase in concentration of this complex leads to its own multimerization, providing a platform for SGs nucleation (Yamagishi et al., 2024). Other proteins, such as TIA1 and G3BP1, as well as untranslated mRNAs, and translation machinery, along with RNA-binding proteins, are recruited to these sites, leading to the formation of cellular organelles without membranes (Costa et al., 2024).

Ataxin-2 has been suggested to be involved in events of endocytosis and cytoskeleton reorganization through the interaction with endophilins A1 and A3, the E3 ubiquitin ligase Cbl, the adaptor Cbl-interacting protein of 85 kDa, and the protein kinase Src. Experimental evidence shows that ataxin-2 delays the internalization of the epidermal growth factor receptor, while its absence accelerates this process. Ataxin-2 also interacts with Parkin, the E3 ubiquitin ligase involved in the endocytic mechanisms, further supporting the involvement of ataxin-2 in this process (Costa et al., 2024).

The role of Ataxin-2 in the calcium homeostasis pathways is suggested to occur through its interaction with the mRNA of regulator of G-protein signaling 8 (RGS8) and the inositol 1,4,5‐trisphosphate receptors (IP3Rs). RGS8 is a protein that inhibits the release of calcium by modulating G-protein coupled receptors. Ataxin-2 stabilizes RGS8 mRNA, regulating its availability and expression. Thus, ataxin-2 can inhibit the release of calcium by preventing the degradation of RGS8 mRNA, maintaining its expression. Ataxin-2 loss of function is believed to disrupt ataxin-2 interaction with RGS8 mRNA, causing it to be degraded, leading to excitotoxity (Costa et al., 2024). Furthermore, it was shown that mutant ataxin-2 interacts with IP3R and exacerbates calcium release from the endoplasmic reticulum. Extensive dendritic arborization and large intracellular calcium stores of Purkinje cells make them highly efficient at neuronal communication but especially vulnerable to calcium fluctuations (Meera et al., 2017).

Another proposed role for ataxin-2 is the modulation of cellular metabolism. In conditions of nutrient availability, mTORC1 promotes anabolic processes. However, in situations of cellular energy deficits, ataxin-2 is upregulated and inhibits mTORC1 by sequestering it into SGs (Costa et al., 2024). By sequestering mTORC1, it reduces its availability and downregulates its downstream effects, shifting cells to a catabolic state. Moreover, knockout models of ataxin-2 exhibit obesity, hepatic steatosis, and insulin resistance, possibly because mTORC1 is unchecked, overactivating the downstream pathway, putting cells into permanent catabolic state. Conversely, the expression of ataxin-2 in the hypothalamus was shown to prevent diet-induced metabolic disorders. Altogether, these observations position ataxin-2 as a metabolic regulator (Costa et al., 2024).

Evidence has also suggested that ataxin-2 is involved in the regulation of the circadian rhythm. In *Drosophila*, the ataxin-2 homolog is known to form complexes with key players of the circadian rhythm (Lsm12 and tyf) to modulate the translation of period mRNA to maintain circadian oscillations in pacemaker neurons. In mammals, *ATXN2* knockout impairs the adaptation to changes in the light/dark cycle in jet-lag models, suggesting a disruption in the circadian rhythm (Costa et al., 2024).

Besides its biological functions in healthy conditions, ataxin-2 is associated with the neurodegenerative disease SCA2 when present in its mutant form. The mutation associated with SCA2 is an abnormal cytosine-adenine-guanine (CAG) trinucleotide expansion within the 1 exon of the *ATXN2* gene (Costa et al., 2024). This mutation is translated into an ataxin-2 form with an abnormal polyQ expansion. In healthy condition, the number of glutamine repeats is lower than 32, whereas in affected individuals, this number is above 34 repetitions (Costa et al., 2024). Multiple cellular mechanisms are disrupted by the presence of the polyQ-expanded ataxin-2, and SCA2 is believed to result from the concerted effects of these impairments. They include protein aggregation, autophagy dysfunction, RNA-mediated toxicity, increased oxidative stress, altered cell signaling, disturbed calcium homeostasis, and changes in SGs dynamics. The disruption of these pathways ultimately results in cerebellar degeneration, with ataxia and slow saccades representing the most characteristic and common motor symptoms observed in patients (Costa et al., 2024).

PolyQ-containing inclusions, characterized by the accumulation of the disease-associated overexpanded protein, are a hallmark of all polyQ diseases, and SCA2 is no exception (Estevam et al., 2023; Costa et al., 2024). In SCA2, mutant ataxin-2 with abnormally long polyQ tracts undergoes structural changes that lead to intracellular inclusions, which are exclusively cytoplasmic in Purkinje cells but can also be intranuclear in pontine nuclei neurons (Costa et al., 2024). Expanded ataxin-2-positive inclusions can sequester proteins involved in key cellular pathways involved in the metabolism, mitophagy, RNA metabolism, protein clearance or SGs formation. Additionally, Intranuclear inclusions, restricted to pontine nuclei neurons, also recruit ataxin-1, ataxin-3, and TBP, proteins related to transcription events, suggesting potential interference with gene expression (Estevam et al., 2023; Costa et al., 2024).

Autophagy impairments are common in polyQ diseases, including SCA2, where the presence of the mutant ataxin-2 leads to the accumulation of autophagy-related proteins such as LC3-II and SQSTM1/p62, indicating defects in autophagosome maturation and clearance (Marcelo et al., 2021b; Costa et al., 2024). These defects have been observed in SCA2 fibroblasts, mouse models, and patient brain tissue. Considering that the UPS has limited ability to clear proteins with abnormal polyQ expansion and the autophagy is impaired in SCA2, along with the high dependence of neurons for the mechanism of clearance, it seems that protein proteostasis is inevitably compromised in SCA2 (Marcelo et al., 2021b; Costa et al., 2024).

Ataxin-2 has also been associated with other diseases, even with glutamine repetitions that are not associated with SCA2. For instance, repetitions of 27–33 CAGs within the *ATXN2* gene have been associated with an increased risk for developing ALS (Van Damme et al., 2011). Although not fully understood, the ALS-SCA2 link could arise from interactions between proteins. In SCA2, TAR DNA-binding protein 43 (TDP-43) is cellularly mislocalized, while in ALS, ataxin-2 shows mislocalization, suggesting a pathological interplay (Costa et al., 2024). Furthermore, under stress conditions, caspase activity is exacerbated in ALS by the presence for intermediate-length ataxin-2, which contributes to an excessive cleavage of TDP-43 into toxic species (Elden et al., 2010). This aligns with evidence linking TDP-43 and ataxin-2 to SGs dynamics – preventing ataxin-2 granule formation mitigates ALS phenotypes in *Drosophila* models (Bakthavachalu et al., 2018). Furthermore, downregulating normal-length ataxin-2 reduces TDP-43-ALS-associated pathology in mouse models (Bakthavachalu et al., 2018).

Similarly, 27–33 CAGs repetitions are associated with an earlier onset of Machado-Joseph disease. Moreover, repetitions of 36–37 CAGs have been associated with atypical cases of Parkinson’s disease (Costa et al., 2024).

Currently, there is no cure or treatment for SCA2. Nevertheless, a preclinical study has shown that antisense oligonucleotides targeting ataxin-2 transcripts are able to both improve Purkinje cell firing and motor performance of mouse models (Scoles et al., 2017). In the future, further experiments must be conducted to better understand how ataxin-2 regulates cellular mechanisms and how it affects other diseases.

*Clévio Nóbrega’s Laboratory is funded by the Cure CSB Project, by the Viljem Julijan Association for Children with Rare Diseases (Slovenia), and by the Algarve Biomedical Center Research Institute (ABC-Ri). AC received a PhD fellowship from the Portuguese Science and Technology Foundation (FCT) #2020.07892.BD.*
